# Long-term all-cause mortality and its association with cardiovascular risk factors in thyroid cancer survivors: an Israeli population-based study

**DOI:** 10.1186/s12885-020-07401-3

**Published:** 2020-09-17

**Authors:** Elena Izkhakov, Lital Keinan-Boker, Micha Barchana, Yacov Shacham, Iris Yaish, Narin N. Carmel Neiderman, Dan M. Fliss, Naftali Stern, Joseph Meyerovitch

**Affiliations:** 1 Institute of Endocrinology, Metabolism and Hypertension, Tel Aviv Sourasky Medical Center, 6 Weizmann Street, 6423906 Tel Aviv, Israel; 2 Sackler Faculty of Medicine, Tel Aviv University, Tel Aviv, Israel; 3 School of Public Health, Faculty of Social Welfare and Health Sciences, University of Haifa, Haifa, Israel; 4 National Cancer Registry, Israel Center for Disease Control, Ministry of Health, Ramat Gan, Israel; 5 Department of Cardiology, Tel Aviv Sourasky Medical Center, Tel Aviv, Israel; 6 Department of Otolaryngology, Head & Neck and Maxillofacial Surgery, Tel Aviv Sourasky Medical Center, Tel Aviv, Israel; 7 Community Division, Clalit Health Services, Tel Aviv, Israel; 8 The Jesse Z. and Sara Lea Shafer Institute for Endocrinology and Diabetes, National Center for Childhood Diabetes, Schneider Children’s Medical Center of Israel, Petah Tikva, Israel

**Keywords:** Thyroid cancer, Mortality, Cardiovascular risk factors

## Abstract

**Background:**

The global incidence of thyroid cancer (TC) has risen considerably during the last three decades, while prognosis is generally favorable. We assessed the long-term all-cause mortality in TC survivors compared to the general population, and its association with cardiovascular risk factors.

**Methods:**

Individuals diagnosed with TC during 2001–2014 (TC group) and age- and sex-matched individuals from the same Israeli healthcare system without thyroid disease or a cancer history (non-TC group) were compared. Cox regression hazard ratios (HRs) and 95% confidence intervals (95%CIs) for all-cause mortality were calculated by exposure status.

**Results:**

During a 15-year follow-up (median 8 years), 577 TC survivors out of 5677 (10.2%) TC patients and 1235 individuals out of 23,962 (5.2%) non-TC patients died. The TC survivors had an increased risk of all-cause mortality (HR = 1.89, 95%CI 1.71–2.10), after adjusting for cardiovascular risk factors already present at follow-up initiation. This increased risk was most pronounced in the 55- to 64-year-old age group (HR = 1.49, 95%CI 1.33–1.67). The TC survivors who died by study closure had more hypertension (14.6% vs. 10.3%, *P* = 0.002), more dyslipidemia (11.4% vs. 7.2%, *P* <  0.001), and more cardiovascular disease (33.6% vs. 22.3%, *P* = 0.05) compared to those who died in the non-TC group.

**Conclusions:**

This large cohort study showed higher all-cause mortality with a higher prevalence of hypertension, dyslipidemia, and cardiovascular disease among TC survivors compared to matched non-TC individuals. Primary and secondary prevention of cardiovascular risk factors in TC survivors is mandatory.

## Background

Thyroid cancer (TC) usually carries an excellent prognosis. Data from the United State Surveillance, Epidemiology, and End Results (SEER) program showed a case fatality rate from TC as being around 0.5 deaths per 100,000 TC survivors [1]. However, a recent analysis of SEER data showed increasing rates of mortality and incidence of TC in the United States from 1974 to 2013, particularly for advanced-stage papillary TC [2]. This is in contrast with an analysis of worldwide data through 2012, which showed a declining mortality from TC in parallel with an increasing incidence [3]. In Israel as well, nationwide trends show an increased incidence of TC and a modest increase in the 5-year relative survival during the last 3 decades [4].

A number of high-risk and pathological conditions have been reported among TC survivors compared to healthy controls, such as an increased prevalence of obesity and diabetes [5], aortic stiffness [6], and isolated left ventricular diastolic dysfunction [7]. Data from the SEER program showed heart disease to be the cause of death for 34% of the non-cancer mortality among TC survivors [8]. Data from our recently published Israeli population-based study showed a 26% increase in cardiovascular and cerebrovascular morbidity among thyroid cancer survivors compared to matched controls, who had neither thyroid disease nor any type of cancer [9]. The risks of all-cause and cardiovascular mortality of differentiated TC (DTC) survivors in The Netherlands, independent of cardiovascular risk factors, were found to be increased by 4.4-fold and by 3.3-fold, respectively, compared to sex- and age-matched controls from the general population in the same region during a median follow-up of 8.5 years [10].

The increased morbidity [5–7] and the increasing incidence of the disease worldwide [3, 11, 12] lends special importance to understanding the elements that comprise the mortality risk of TC survivors.

The aims of the current study, therefore, were 1) to assess the long-term (15-year) all-cause mortality in a large cohort of Israeli TC survivors compared to members of the same healthcare services who had neither thyroid disease nor a diagnosis of cancer; 2) to investigate the association of long-term all-cause mortality with cardiovascular risk factors among those TC survivors.

## Methods

### Study design

This large historical cohort study (as previously described elsewhere) [9] is based on the computerized data of the Clalit Health Services (CHS), the largest healthcare fund in Israel and a provider of healthcare to more than 4.3 million Israeli residents (52.3% of the total Israeli population). The CHS database comprises demographic and medical data, including diagnoses, laboratory tests, drug prescriptions and purchases, and date (but not cause) of death.

### Study population and follow-up

The TC survivors (TC group) included individuals diagnosed with TC between January 1, 2001 and December 31, 2014, who underwent thyroidectomy and received radioactive iodine treatment and levothyroxine therapy. The non-TC group was comprised of CHS members who had neither thyroid disease nor any type of cancer during the same time period. The non-TC group was matched to the TC group by sex and age (± 2 years) at a ratio of 4:1. The follow-up of the TC survivors started on the date of TC diagnosis, and the follow-up of the matched non-TC individuals started in the same year of diagnosis as the TC survivors to whom they were matched. The follow-up of both groups ended on June 30, 2016 or on the date of death, whichever occurred earlier.

The Medical Ethics Committee of the CHS provided approval to conduct this study. Signed informed consent from the participants was waived since the study was based on existing databases. The study inclusion criteria were membership in the CHS throughout the entire study period (January 1, 2001 to June 30, 2016) and age ≥ 18 years. The exclusion criteria were any other primary cancer prior to study entry, with the exception of squamous or basal cell carcinoma of the skin, and advanced renal failure (creatinine > 1.5 mg/dL), the latter because of its impact on the clinical decisions regarding the application of radioactive iodine treatment.

### Study variables

The following data were collected for the TC and non-TC groups: demographic characteristics, smoking status, anthropometric characteristics, mean blood pressure, pulse rate, and laboratory tests that included levels of glucose and creatinine, lipid profile, and thyroid function tests at a date closest to study entry, and any diagnosed pathological conditions at study entry and at the end of the follow-up.

Diabetes mellitus was defined as having at least one of the following upon study entry: a diagnosis of diabetes mellitus recorded in the CHS database, 2 plasma glucose measurements > 125 mg/dL, a random plasma glucose measurement > 199 mg/dL, or a record of hypoglycemic medications. Hypertension was defined as having at least one of the following upon study entry: a diagnosis of hypertension recorded in the CHS registry, 3 or more measurements of systolic blood pressure > 140 mmHg or diastolic blood pressure > 90 mmHg, or a record of medications for hypertension. Dyslipidemia was defined as having at least one of the following upon study entry: a diagnosis of hyperlipidemia recorded in the CHS registry, at least 2 plasma low density lipoprotein (LDL) cholesterol measurements > 160 mg/dL, a triglyceride level > 150 mg/dL, or a high-density lipoprotein cholesterol level < 40 mg/dL for males or < 50 mg/dL for females, or a record of hypolipidemic medications. Cardiovascular disease was defined by the International Statistical Classification of Diseases and Related Health Problems (ICD) code as having at least one of the following diagnoses recorded in the CHS database upon study entry: ischemic heart disease, acute myocardial infarction, percutaneous transluminal coronary angioplasty, or coronary artery bypass graft. Cerebrovascular disease was defined by ICD code as having at least one of the following diagnoses recorded in the CHS database code upon study entry: transient ischemic attack, cerebrovascular accident, carotid artery stenosis/occlusion, or carotid endarterectomy. The prevalence of diabetes mellitus, hypertension, dyslipidemia, and cardiovascular and cerebrovascular diseases at the end of follow-up was assessed according to the same definitions as those for the beginning of follow-up.

### Statistical analyses

Baseline patient characteristics are presented as means and standard deviations for continuous variables, and as frequencies and percentages for categorical variables. Independent t-tests and Chi-square tests were used to compare between the study groups for continuous and categorical variables, respectively. Unadjusted and adjusted Cox proportional hazard models were performed to evaluate the hazard ratios (HRs) and 95% confidence intervals (95%CIs) for death. Kaplan-Meier-based adjusted survival curves for mortality are provided. All-cause mortality risk was compared between the TC and the non-TC groups by sex and age (≤44, 45–54, 55–64, 65–74, and ≥ 75 years) at study entry. Unadjusted and adjusted Cox proportional hazard models were performed to evaluate the HRs and 95%CIs for long-term mortality by a minimum latency period of 2 years. Unadjusted and adjusted Cox proportional hazard models were performed to evaluate the HRs and 95%CIs for long-term mortality among the TC survivors by the number of cardiovascular risk factors (hypertension, dyslipidemia, diabetes mellitus, cardiovascular or cerebrovascular disease) at the end of the follow-up period. Given the age difference between the TC and the non-TC groups, all of the adjusted Cox proportional hazard models included age and sex in addition to other relevant variables. Data were analyzed with SPSS software version 23.0. (SPSS Inc. Headquarters, 233 S. Wacker Drive, 11th floor Chicago, Illinois 60,606, USA). A 2-sided *P* value less than 0.05 was considered statistically significant.

## Results

### Baseline characteristics of the TC and non-TC groups

The TC group was comprised of 5677 TC survivors (mean age 50 ± 16 years) of whom 21.4% were males. The non-TC group was comprised of 23,962 members of CHS (mean age 47 ± 15 years) of whom 20.5% were males. Although the study sample was matched for age and sex, exclusion of individuals with renal failure slightly changed the distributions (Table 1) [9]. At baseline, the anthropometric characteristics, mean blood pressure, and pulse rate were similar between the study and control groups (Table 1) [9]. A higher proportion of individuals in the TC group had hypertension compared to the non-TC group (24.7% vs. 19.3%, respectively, *P* < 0.001) and dyslipidemia (32.9% vs. 28.5%, *P* < 0.001), and a lower proportion had cardiovascular and cerebrovascular diseases (1.4% vs. 4.9 and 0.5% vs. 1.7%, *P* < 0.001 for both) (Table 2) [9].

**Table 1:** Baseline characteristics of the thyroid cancer survivors (*n* = 5677) and non-thyroid cancer individuals (*n* = 23,962)

Characteristic	Cancer group (*n* = 5677)	Non-cancer group (*n* = 23,962)
Male sex, *n* (%)	1216 (21.4)	4912 (20.5)
Age, years, mean ± SD	50 ± 16	47 ± 15
Median (range)	49 (18–108)	46 (17–100)
Smoking		
Past, *n* (%)	371 (6.5)	1249 (5.2)
Current, *n* (%)	947 (16.7)	6080 (25.4)
Weight, kg, mean ± SD	78 ± 21	76 ± 21
Height, cm, mean ± SD	164 ± 8.7	163 ± 8.6
BMI, kg/m^2^, mean ± SD	28.5 ± 6.49	28.0 ± 6.8
< 18.5, *n* (%)	38 (1.4)	256 (2.0)
18.5–24.99, *n* (%)	820 (30.9)	4527 (34.7)
25–29.99, *n* (%)	893 (33.7)	4221 (32.4)
≥30, *n* (%)	900 (33.9)	4031 (30.9)
SBP, mm Hg, mean ± SD	123 ± 15	122 ± 16
DBP, mm Hg, mean ± SD	75.4 ± 9.0	74.6 ± 10.6
Pulse, beat/min, mean ± SD	76 ± 10.2	76 ± 10.4

*SD* Standard deviation, *BMI* Body mass index, *SBP* Systolic blood pressure, *DBP* Diastolic blood pressure

**Table 2:** Baseline morbidity of the thyroid cancer survivors (*n* = 5677) and non-thyroid cancer individuals (*n* = 23,962)

Variable	Cancer group (*n* = 5677)	Non-cancer group (*n* = 23,962)	*P*
Hypertension^a^, n (%)	1405 (24.7)	4629 (19.3)	**< 0.001**
Diabetes mellitus^b^, n (%)	707 (12.5)	2681 (11.2)	**0.007**
Dyslipidemia^c^, n (%)	1865 (32.9)	6822 (28.5)	**< 0.001**
AF, *n* (%)	170 (0.3)	117 (0.5)	**< 0.001**
Rheumatic heart disease, *n* (%)	8 (0.1)	16 (0.1)	0.077
Fatty liver, *n* (%)	101 (1.8)	285 (1.2)	**< 0.001**
Cerebrovascular disease^*^1, *n* (%)	53 (0.9)	624 (2.6)	**< 0.001**
Cardiovascular disease^*^2, *n* (%)	119 (2.1)	1330 (5.6)	**< 0.001**
Cerebrovascular & cardiovascular diseases ^*^1 & ^*^2, *n* (%)	154 (2.7)	1.631 (6.8)	**< 0.001**

^a^Physician diagnosis of hypertension or at least three measurements of systolic blood pressure > 140 mmHg, or of diastolic blood pressure > 90 mmHg, or hypertension medications; ^b^Physician diagnosis of diabetes mellitus, or twice blood fasting glucose ≥126 mg/dL, or random blood glucose ≥200 mg/dL, or hypoglycemic medications; ^c^Physician diagnosis of hyperlipidemia, or at least two measurements of LDL > 160 mg/dL, or TG > 150 mg/dL, or HDL < 40 mg/dL for males, or HDL < 50 mg/dL for females, or hypolipidemic medications. AF, Atrial fibrillation; ^*^1, Transient ischemic attack, cerebral vascular attack, carotid artery stenosis and occlusion, carotid endarterectomy; ^*^2, Ischemic heart disease, acute myocardial infarction, percutaneous transluminal coronary angioplasty, coronary artery bypass graft

**Bold** indicates significant

### Mortality in the TC and non-TC groups

**Fig. 1:**
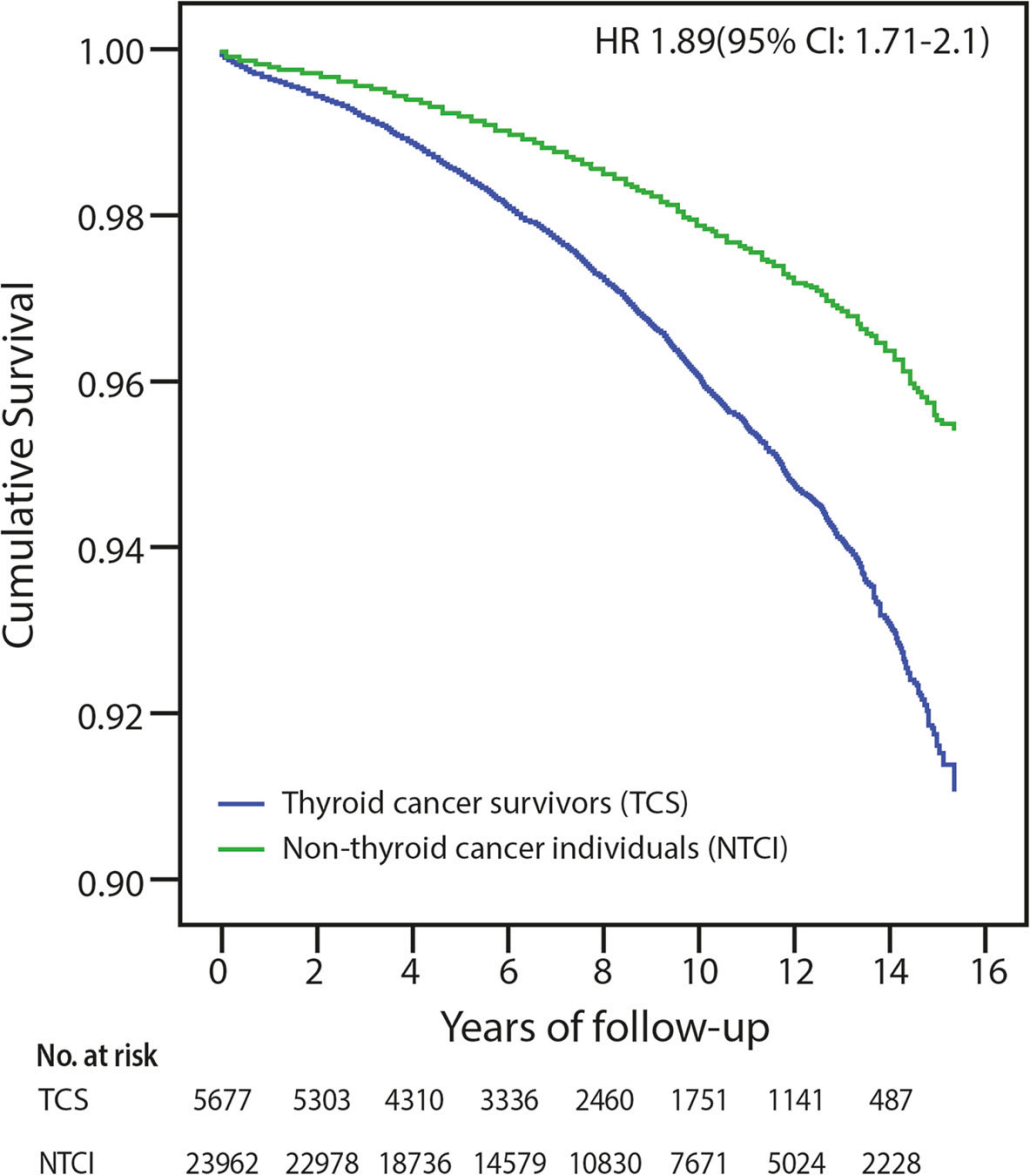
Kaplan-Meier survival curves for the thyroid cancer survivors (*n* = 5677) and non-thyroid cancer individuals (*n* = 23,962). The survival curve is steeper for the thyroid cancer (blue) than for the non-cancer (green) groups. The data were adjusted for age, sex, cerebrovascular and cardiovascular disease, hypertension, diabetes mellitus, dyslipidemia, and current smoking

During the study period, a total of 1812 participants died, of whom 577 (10.2%) were in the TC group and 1235 (5.2%) in the non-TC group. The mean survival for those who died was 7.6 ± 4.2 and 8.0 ± 4.1 years, respectively. All-cause mortality for the entire cohort was associated with being older (HR 1.11; 95%CI: 1.10–1.11), having a diagnosis of hypertension (HR 1.15; 95%CI: 1.02–1.29) or diabetes mellitus (HR 1.68; 95%CI: 1.52–1.86), or having a previous cerebrovascular disease (HR 1.39; 95%CI: 1.16–1.68) or a cardiovascular disease (HR 1.31; 95% CI: 1.09–1.56), and current smoking (HR 1.32; 95% CI: 1.17–1.50). Female sex and a diagnosis of dyslipidemia were associated with a lower risk for mortality (HR 0.70; 95%CI: 0.63–0.78 and HR 0.85; 95%CI: 0.76–0.94, respectively). The mean baseline glucose and LDL levels were higher among those who died than among those who survived (120 ± 52 vs. 90 ± 29, *P* < 0.001 and 199 ± 43 vs. 193 ± 38, *P* < 0.001, respectively). In the univariate analysis, the HR for mortality in the TC group compared to the non-TC group was 2.03 (95%CI: 1.84–2.24). In a model adjusted for age and sex, the HR for mortality was 1.78 (95%CI: 1.61–1.96) for the TC group compared to the non-TC group. The HR for mortality in the TC survivors compared to the controls was even stronger, i.e., HR 1.89 (95%CI: 1.71–2.10), after further adjustment for age, sex, prevalence of cerebrovascular and cardiovascular disease, hypertension, diabetes mellitus, dyslipidemia, and current smoking at the time of study onset. The Kaplan-Meier survival curve of the TC group was steeper than that of the non-TC group (Fig. 1). After stratifying by sex and adjusting for age and for the prevalence of cerebrovascular and cardiovascular disease, hypertension, diabetes mellitus, dyslipidemia and current smoking at study onset, the HR for mortality was higher for the male TC survivors (HR 1.73, 95%CI: 1.44–2.08) than for the non-TC males (HR 1.29, 95%CI: 1.14–1.47).

The adjusted HRs for mortality were significantly higher for the TC group compared to the non-TC group in all age groups at the end of the same follow-up time period, with the 55- to 64-year age group showing the highest values (HR = 1.49, 95%CI: 1.33–1.67) (Table 3). Stratification by follow-up period in the TC group showed the following time distribution of mortality events: 7.1% in years 0–2 after TC diagnosis, 25.8% in years 2–5, 36.2% in years 5–10, and 30.9% in > 10 years.

**Table 3:** Cox proportional hazard ratios of mortality in the thyroid cancer survivors (*n* = 5677) and non-thyroid cancer individuals (*n* = 23,962) by age groups, crude and adjusted for covariates^a^

Age	Crude HR for death in the cancer vs. non-cancer groups (95%CI)	*P* value	Adjusted HR for death in the cancer vs. non-cancer groups (95%CI)	*P* value
≥44	1.49 (1.25–1.77)	**< 0.001**	1.33 (1.06–1.67)	**< 0.05**
45–54	1.34 (0.96–1.89)	0.088	1.25 (1.06–1.48)	**< 0.05**
55–64	2.02 (1.61–2.54)	**< 0.001**	1.49 (1.33–1.67)	**< 0.001**
65–74	1.62 (1.35–1.95)	**< 0.001**	1.33 (1.21–1.46)	**< 0.001**
75+	1.49 (1.25–1.77)	**< 0.001**	1.35 (1.24–1.47)	**< 0.001**

*HR* Hazard ratio, *CI* Confidence interval

^a^Adjusted for sex, cerebrovascular and cardiovascular disease, hypertension, diabetes mellitus, dyslipidemia, and current smoking

**Bold** indicates significant

The mortality events that occurred during the first 2 years of follow-up were excluded in order to allow a minimal latency period and to assess long-term mortality. The subsequent HR for mortality in the TC group adjusted for prevalent cardiovascular risk factors (age, sex, hypertension, diabetes mellitus, dyslipidemia, and current smoking) and for prevalent atherosclerotic cardiovascular and cerebrovascular disease at follow-up onset remained significantly increased (HR 1.59, 95%CI: 1.40–1.80).

### Association between cardiovascular risk factors and long-term all-cause mortality

Compared to the individuals in the non-TC group who died, the TC survivors who died had a higher prevalence of hypertension (14.6% vs. 10.3%, respectively, *P* = 0.002), dyslipidemia (11.4% vs. 7.2%, *P* < 0.001), and cardiovascular disease (33.6% vs. 22.3%, *P* < 0.001) at the end of the 15-year follow-up period. Stratification of the TC patient group by selected cardiovascular risk factors (hypertension, dyslipidemia, diabetes mellitus, and cardiovascular and cerebrovascular disease) at the end of the follow-up period revealed a direct association between the number of those risk factors and mortality risk (Table 4). The adjusted HRs for mortality in the TC group in the presence of two, three or four cardiovascular risk factors were significantly elevated (HR 1.23, 95%CI: 1.38–1.99; HR 1.66, 95%CI: 1.38–1.99; HR 2.59, 95%CI: 2.11–3.19, respectively).

**Table 4:** Cox proportional hazard ratios of mortality in the thyroid cancer survivors (*n* = 5677) by number of cardiovascular risk factors (hypertension, dyslipidemia, diabetes mellitus, cardiovascular or cerebrovascular disease) at the end of the follow-up period, crude and adjusted for covariates^a^

Number of CV RF	Crude HR (95%CI)	*P* value	Adjusted HR (95%CI)	*P* value
1	1.27 (0.94–1.70)	0.12	0.97 (0.81–1.16)	0.72
2	2.89 (2.19–3.81)	**< 0.001**	1.23 (1.03–1.47)	**0.02**
3	4.86 (3.65–6.47)	**< 0.001**	1.66 (1.38–1.99)	**< 0.001**
4	6.73 (4.71–9.63)	**< 0.001**	2.59 (2.11–3.19)	**< 0.001**

*CV RF* Cardiovascular risk factors, *HR* Hazard ratio, *CI* Confidence interval

^a^Adjusted for age and sex

**Bold** indicates significant

### Long-term all-cause mortality and new onset malignancy

During the study period, second primary tumors among the TC survivors who died were not more common than primary tumors among the individuals in the non-TC group who died.

## Discussion

### Overall all-cause mortality risk

This large Israeli population-based historical cohort study demonstrated a higher all-cause mortality rate among TC survivors than among individuals without any thyroid disease or any type of cancer from the same population, matched by sex and age. The difference remained statistically significant after adjusting for age, sex, baseline cardiovascular risk factors, and cardiovascular and cerebrovascular diseases at study onset. The novel findings of this study are the association between cardiovascular risk factors at the end of follow-up and the higher mortality among Israeli TC survivors compared to non-TC individuals. In contrast, there was no association between mortality and the presence of a second primary malignancy in the former group and the presence of a primary malignancy in the latter group at the end of follow-up.

The majority of the TC survivors in the current cohort were female (79%). Stratification by sex showed that the mortality HR for male TC survivors was higher than that of females. Similarly, analysis of the SEER data revealed a higher mortality rate among male TC survivors than among females [2]. Moreover, during the last three decades, age-adjusted mortality attributed to TC was reported to remain stable among Israeli men and to decrease among Israeli women [4].

Interestingly, there were nearly 10% more current smokers in the non-TC group compared with the TC group. That inverse association between smoking and the development of TC was present in both females (HR = 0.54, 95% CI: 0.35–082) and males (HR = 0.31, 95% CI: 0.09–1.04), suggesting that smoking may be a protective factor, as observed by Meinhold et al. [13]

Surprisingly, female sex and a diagnosis of dyslipidemia at the beginning of the follow-up for the entire cohort were associated with a lower risk for mortality. This finding may be explained at least in part by statin treatment.

### Mortality risk by age

There was a significant and pronounced age-related increased mortality risk for TC survivors compared to the non-TC group, particularly from age 55 years onwards. Our findings concur with a recent study that found 55 years to be a valid cutoff age for risk assessment in TC survivors [14]. Another recent publication concluded that the increasing age risk should be considered along a continuum [15]. The SEER study also reported that the 5-year and 10-year probability of death increased with age among TC survivors [8]. The lower relative mortality risk among younger TC survivors is supported by the results of a German study that disclosed no reduced life expectancy among DTC survivors who were under 45 years of age at DTC diagnosis and with tumor-node-metastasis (TNM) stages I, II, or III compared to the general population [16].

### Factors contributing to increased mortality among TC survivors

The interpretation of our results should take into account the various factors that may contribute to increased mortality in TC survivors. First, despite the excellent prognosis and the low and decreasing fatality rates, the small proportion of TC survivors who die of their disease naturally increases the general mortality rate in TC survivors, particularly in the long term during which recurrence is a factor. In a Dutch study [10], progression or recurrence of TC was the cause of mortality for 39% of TC survivors who died during a mean follow-up of 8.5 years. An increased risk of second primary cancers, compared to the general population, may also increase overall mortality. Such risk has been documented in Israel [17] and elsewhere [18]. Although a second primary cancer also worsens the prognosis of TC survivors [19], a second primary cancer was not more common among the TC survivors who died than a de novo primary cancer among the individuals who died in the non-TC group in the current study. However, analyses of the current findings revealed a direct association between the number of cardiovascular risk factors and mortality risk among the TC patients.

Increased mortality due to non-cancer causes must also be considered in relation to TC. SEER data showed that the risk of dying from a cause other than the primary disease in TC survivors is nearly 2-fold than that of dying from TC [8]. In that study, the 10-year probabilities of death from TC, from other cancers, and from non-cancer causes were 3.0, 2.0 and 3.9%, respectively. In the current study, increased mortality persisted after controlling for baseline cardiovascular risk factors and morbidity, in addition to age and sex. At the time of TC diagnosis, the anthropometric and clinical characteristics of the TC survivors were very similar to those of the non-cancer individuals, with the exception of hypertension, which was slightly more prevalent among the TC survivors. Similarly, a Dutch population-based study reported a higher than expected prevalence of hypertension among TC survivors [20]. In the current study, the prevalence of diabetes mellitus at baseline was similar between the TC group and the non-TC group. This concurs with a recently published Israeli study that was based on a nationwide cohort and which found no association between diabetes mellitus and TC [21]. Nevertheless, diabetes mellitus may still affect TC prognosis. For example, TC survivors with type 2 diabetes mellitus and DTC were found to be more likely to have an advanced TNM stage at the time of diagnosis as well as increased disease-specific mortality [22]. The prevalence of hypertension, dyslipidemia, and cardiovascular disease at the end of the follow-up were higher for the individuals in the TC group who died during the follow-up period of the current study than among those in the non-TC group who died. These differences between the groups at study closure were greater than those recorded at baseline.

The treatment of TC may affect the mortality risk in a number of ways. The majority of the Israeli TC survivors were treated according to a standard of care consisting of thyroidectomy, radioactive ablation, and thyroid hormone suppression treatment. Several studies have shown a greater risk of second primary cancer among TC survivors treated with radioactive ablation than among those who did not undergo such treatment [18, 19]. This increased risk was shown to also prevail among low-risk TC survivors [23] and only when the cumulative radioactive iodine dose was ≥37.0 GBq [24]. The mortality risk was also higher for the 5–15% of TC survivors who become refractory to radioactive iodine therapy [25, 26].

Thyroid hormone suppression is a component of TC treatment, both for those who respond and for those who are refractory to radioactive iodine therapy. Associations have been reported among thyroid hormone suppression treatment and atrial fibrillation [27], impaired small and large artery elasticity [28], increased left ventricular mass [28], abnormalities of heart morphology related to impaired exercise performance [29], a prothrombotic condition [30], and myocardial strain [31]. In the absence of data on thyroid hormone treatment, we do not know the degree to which subclinical hyperthyroidism was achieved in the TC survivors, or the proportion of individuals in the TC group that may have had endogenous subclinical hyperthyroidism. However, subclinical hyperthyroidism not in the setting of TC has been shown to be associated with atrial fibrillation [32, 33] worse physical capacity [34], increased risk of heart failure, and cardiovascular morbidity [33, 35]. Moreover, endogenous subclinical hyperthyroidism was associated with increased risks of total and cardiovascular mortality in a pooled analysis from 10 cohorts of individuals not treated with thyroxine [32]. A Danish population-based study found increased heart failure and increased cardiovascular and all-cause mortality among patients with subclinical hyperthyroidism compared to euthyroid individuals [36]. In contrast with these reports, a recently published small study with up to a 9-year follow-up showed no impairment in cardiac function and structure among individuals who received thyroid hormone suppression treatment [37]. However, the patients’ thyroid-stimulating hormone (TSH) levels in that study were < 0.1 mU/L in the intermediate-risk-of-recurrence group and < 0.3 mU/L in the lower-risk-of-recurrence group, which is higher than the recommendations for TSH suppression. Having no data on TSH levels during the follow-up in our cohort, we were unable to compile the effects of treatment on mortality.

Health-related quality of life (HRQoL) is an additional factor that may affect health and long-term mortality in cancer survivors. A recent publication reported that 14–17 years after diagnosis, almost half of DTC survivors who filled in a HRQoL questionnaire expressed anxiety about disease recurrence, and that this negatively impacted their HRQoL [38]. Poor HRQoL has been found to be related to all-cause mortality in various populations [39, 40].

Stage at diagnosis [2, 16] and genetic variance [41] have been shown to affect mortality. The current study did not distinguish between types of TC. DTC, which comprised the vast majority of TCs in our population, confers considerably better prognosis than do the less common types of TC [1].

The strengths of this study are the long-term follow-up findings of a large population-based cohort, which are based on the computerized data file of the largest healthcare fund in Israel (covering around 52.3% of the total Israeli population). They take into account baseline pathological conditions and those diagnosed during the follow-up period of the study (including dyslipidemia, hypertension, diabetes mellitus, cardiovascular and cerebrovascular diseases, and new malignancies). A significant limitation of this study is the lack of data on the cause of death, as well as on other variables, such as the histological variant of the TC, the stage of the TC, the doses of radioactive iodine treatment, and the TSH levels during the follow-up period.

## Conclusions

The finding of a higher all-cause mortality risk in TC survivors compared to the general population, despite the excellent prognosis and the decreasing fatality rates for TC, is of concern. In the current study, a higher prevalence of hypertension, dyslipidemia, and cardiovascular disease at the end of the follow-up period was associated with mortality among the group of Israeli TC survivors. Moreover, we found a direct association between the number of cardiovascular risk factors at the end of the follow-up and the mortality risk among TC survivors. As such, our findings suggest that cardiovascular risk factors may predict higher mortality in TC survivors compared to non-TC individuals. We therefore recommend a high level of awareness of cardiovascular risk factors, their follow-up, and their treatment among TC survivors, with the aim of reducing the risk of mortality among them.

## Data Availability

The data that support the findings of this study are available from database of Clalit Health Services, but restrictions apply to the availability of these data, which were used under license for the current study, and so are not publicly available. Data are however available from the authors upon reasonable request and with permission of Clalit Health Services.

## Abbreviations

TC: Thyroid cancer
HR: Hazard ratio
CI: Confidence interval
SEER: United State Surveillance, Epidemiology, and End Results
DTC: Differentiated thyroid cancer
CHS: Clalit Health Services
LDL: Low density lipoprotein
ICD: International Statistical Classification of Diseases and Related Health Problems
TNM: Tumor-node-metastasis
TSH: Thyroid-stimulating hormone
HRQoL: Health-related quality of life
SD: Standard deviation
BMI: Body mass index
SBP: Systolic blood pressure
DBP: Diastolic blood pressure.
AF: Atrial fibrillation
CV RF: Cardiovascular risk factors
